# Cannabis Use and Clinical Outcomes in First Episode Psychosis: Results From a 2‐Year Follow‐Up Study

**DOI:** 10.1111/eip.70179

**Published:** 2026-05-16

**Authors:** Ilaria Domenicano, Alice Onofrio, Domenico De Donatis, Sara Malservigi, Giuseppe Di Vincenzo, Martino Belvederi Murri, Luigi Grassi, Maria Ferrara

**Affiliations:** ^1^ Institute of Psychiatry, Department of Neuroscience and Rehabilitation University of Ferrara Ferrara Italy; ^2^ Integrated Department of Mental Health and Pathological Addictions, Ferrara Health Trust Ferrara Italy; ^3^ Department of Psychiatry Yale University School of Medicine New Haven Connecticut USA

**Keywords:** cannabis use, clinical outcomes, first episode psychosis, recovery, untreated psychosis

## Abstract

**Aim:**

This study aimed to investigate the association between cannabis use prior to enrollment in a First Episode Psychosis (FEP) program and prognosis, including severity of psychiatric symptoms, type of antipsychotic treatment prescribed, and clinical recovery.

**Methods:**

A longitudinal cohort study was conducted on patients aged 18–35 diagnosed with affective and non‐affective psychosis who were referred to the FEP Program in Ferrara, Italy, between 2012 and April 2024. Sociodemographic and clinical data were collected at program entry and every six months for up to 24 months. Cannabis use was categorised dichotomously based on reported or charted use within the month prior to program entry. The Health of the Nation Outcome Scale (HoNOS) was used to monitor clinical outcomes (assessed through reductions of the total HoNOS score, employment status, and type of antipsychotic prescription) and recovery (total HoNOS < 8). Bivariate analyses compared sociodemographic characteristics and clinical outcomes between people with cannabis use and people without cannabis use at baseline and at 1‐ and 2‐year follow‐ups, considering both baseline cannabis status and current cannabis use status. A Mixed Model for Repeated Measures, was used to compare the average HoNOS scores and the recovery rates between the two groups at several endpoints (baseline, 12 months and 24 months).

**Results:**

The total sample included 173 patients, predominantly men (74.5%), diagnosed with non‐affective psychosis (83.8%), with a median age at onset of psychotic symptoms of 22.9 years. At admission, individuals reporting cannabis use (48%), compared to those without cannabis use, were predominantly male (87.9% vs. 62.2%, *p* < 0.001), had a significantly shorter duration of untreated psychosis (32.8 vs. 52.2 weeks, *p* = 0.05) and were more likely to receive combined oral and depot antipsychotic treatment (16.9% vs. 5.6%, *p* = 0.05), no difference in baseline HoNOS scores was observed. At 1‐ and 2‐year follow‐ups, no significant differences in overall psychopathology were found between groups under either cannabis use condition. However, baseline cannabis exposure was associated with lower rates of student status at both 1‐year (11.8% vs. 24.3%) and 2‐year (6.3% vs. 23.2%) follow‐ups. At the 1‐year follow‐up, participants with cannabis use were more likely to receive a combination of long‐acting injectable (LAI) and oral antipsychotics (21.1% vs. 7.3%, *p* = 0.05) compared to those without cannabis use. No significant differences were found between the two groups in terms of HoNOS scores or recovery rates at any time point (baseline, 12 months, 24 months).

**Conclusions:**

Cannabis use prior to FEP program entry was not associated with significant differences in clinical outcomes or recovery, despite being admitted with a shorter DUP and being predominantly male. Nevertheless, cannabis use was associated with more intensive antipsychotic treatment, both at admission and at 1 year. These findings underscore the importance of early detection of cannabis use and integrated care addressing both psychiatric symptoms and substance use. Future randomised controlled trials should explore whether reducing cannabis use can decrease antipsychotic burden and improve long‐term outcomes.

## 1 Introduction

Cannabis is the most commonly used psychoactive substance worldwide and ranks as the third most addictive substance after alcohol and tobacco (Fischer et al. 2022). Among its various components, delta‐9‐tetrahydrocannabinol (THC) is the primary psychoactive compound capable of crossing the blood–brain barrier. THC is associated with a range of psychotic symptoms, including suspiciousness, paranoid delusions, euphoria, disorganised thinking, and perceptual disturbances (Belvederi Murri et al. 2025; D'Souza et al. 2016).

Over the past two decades, increasing research attention has been devoted to the potential causal relationship between cannabis use and psychiatric disorders, particularly the onset of psychosis (McLaren et al. 2010). Cannabis use has been identified both as a risk factor associated with the development of psychotic disorders—especially in individuals who consume high‐potency cannabis frequently (Di Forti et al. 2019)—and as a negative prognostic indicator (Andréasson et al. 1987; Marconi et al. 2016; Hjorthøj et al. 2021; Ricci et al. 2023). Notably, individuals who use high‐THC cannabis are more likely to show poor adherence to antipsychotic medication and experience more frequent relapses (Foglia et al. 2017); compared to traditional cannabis, the use of synthetic cannabinoids is associated with significantly more frequent emergency department visits, with users being up to 30 times more likely to require urgent care (Winstock et al. 2015). A multicenter case–control study conducted across 11 European cities, examining 901 patients experiencing a first episode of psychosis (FEP) and 1237 controls, estimated that eliminating high‐potency cannabis use could prevent up to 12% of FEP cases across these sites, with even higher preventable fractions in cities like London (30%) and Amsterdam (50%) (Di Forti et al. 2019).

Several studies have also aimed to characterise cannabis‐induced psychosis in greater detail. Epidemiological findings suggest that this disorder is more prevalent among males and tends to appear earlier in younger individuals (Ochoa et al. 2012). Furthermore, consistent evidence indicates that daily cannabis use before the age of 15 is linked to an earlier onset of psychotic symptoms (Di Forti et al. 2014; Chadwick et al. 2013; Arseneault et al. 2002). Kline et al. (2022) further demonstrated that early cannabis exposure correlates with earlier onset of both clinical high‐risk states and full psychosis, poorer premorbid functioning, and greater severity of cannabis use disorder at the time of presentation. Since an earlier age of onset is a well‐established predictor of worse clinical prognosis in psychotic disorders (Tosato et al. 2013), these findings support the hypothesis that cannabis use influences FEP outcomes.

In addition, individuals with psychosis who use cannabis have been shown to report more dissociative experiences (Ricci et al. 2023) and increased severity of psychotic symptoms, both of which are associated with elevated risks of suicidality, aggressive behaviour, and legal problems (Togay et al. 2015; Rolin et al. 2019). Kejser Starzer et al. (2018) conducted a large‐scale registry‐based study in Denmark that tracked 6788 individuals diagnosed with substance‐induced psychosis between 1994 and 2014: they found that cannabis‐induced psychosis had the highest rate of conversion to schizophrenia or bipolar disorder (47.4%), with younger age being a significant risk factor for conversion. Half of the transitions to schizophrenia occurred within 3.1 years, and half of the transitions to bipolar disorder occurred within 4.4 years of the initial diagnosis.

Collectively, the literature indicates that cannabis use is a significant negative prognostic factor in FEP. It is associated with worse clinical presentation, functional outcomes (Large et al. 2014), increased rates of hospitalisation (Schoeler et al. 2016, 2017a), lower treatment adherence (Miller et al. 2009; Faridi et al. 2012), more frequent and earlier relapses, particularly among those using high‐potency cannabis (Schoeler et al. 2017a; Ricci et al. 2023), and reduced antipsychotic treatment effectiveness (Schoeler et al. 2017b).

In Italy, recent data from the 2023 Annual Report to Parliament on Drug Addiction revealed a concerning increase in cannabis use among adolescents (Presidenza del Consiglio dei Ministri Dipartimento per le Politiche Antidroga 2023). The prevalence of use among young people rose from 18.7% in 2021 to 27.9%. Approximately 4 million Italians aged 18–84 (8.5%) reported using cannabis at least once during the year, and wastewater analysis estimated about 50 daily doses per 1000 residents. Alarmingly, over 580000 students aged 15–19 (24%) reported cannabis use in the previous year, returning to pre‐pandemic prevalence levels. Given that the young adult age group also coincides with the peak onset period for FEP, understanding the role of cannabis use in the clinical presentation and prognosis of FEP is of critical importance (Allegri et al. 2013; Belvederi Murri et al. 2021; Ferrara et al. 2022; Ferrara et al. 2024; Tarricone et al. 2012).

In this context, the present cross‐sectional study aims to evaluate associations between cannabis use prior and after admission to a specialised FEP program in Ferrara, Northern Italy, on clinical outcomes at one and two years. Specifically, it investigates the relationship between cannabis exposure and overall symptom severity, antipsychotic treatment patterns, and rates of clinical recovery.

## 2 Methods

### 2.1 Setting

Participants were those consecutively admitted to the FEP program in Ferrara from 2012 to April 2024. The FEP programme (FES) is provided by the outpatient community psychiatric services (CMHC) within the Integrated Department of Mental Health and Pathological Addictions (IDMHPA) according to a “specialist within generalist” model (Ferrara, Tedeschini, et al. 2019) to individuals aged 15–35 with a diagnosis of affective and non‐affective FEP or at high risk of developing FEP (clinical high risk for psychosis—CHR) (Belvederi Murri et al. 2023; Ferrara et al. 2022).

Specifically, the FES provides pharmacological interventions, cognitive‐behavioural therapy, psycho‐educational interventions for both patients and their families, and social inclusion programs. These multi‐dimensional interventions aim to reduce the Duration of Untreated Psychosis (DUP) by facilitating immediate screening of suspected cases, prompt initiation of pharmacological treatment, and outreach to potential referral services, thereby promoting the widest and earliest recovery (Gruppo di lavoro Esordi Psicotici, Regione Emilia‐Romagna 2016). The FEP‐PDTA (Percorso diagnostico terapeutico assistenziale—diagnostic and therapeutic care pathway) includes the collaboration with the Service for Addiction Disorders (SerD), which is a community outpatient service for patients with addiction disorders. It offers outpatient medical care, including physical health monitoring, social service interventions, and psychological interventions. Moreover, the SerD can also monitor substance use by periodic urine and blood tests. Therefore, the psychiatrist within the FEP‐PDTA can refer patients with ongoing substance abuse to the SerD to collaborate in providing psychoeducational, motivational interventions, and pharmacological treatment.

Patients receive care by the FES for up to three years, according to their clinical needs. At discharge, either standard care at CMHC is provided or users are referred to their general practitioner. Referral to the program can occur through direct access to CMHCs, the psychiatric inpatient unit, psychologists/social services, and general practitioners (Belvederi Murri et al. 2021).

### 2.2 Participants

According to the local PDTA, inclusion criteria for the FES are as follows: (a) age between 15 and 35 years; (b) having been in treatment for a psychotic disorder for no longer than 24 months; (c) diagnosis of affective and non‐affective psychosis; or (d) being diagnosed as at‐risk mental state for psychosis (CHR). Patients are excluded from the program if they have the following: (a) a severe intellectual disability (IQ < 50); (b) psychosis induced by a medical condition.

This study included all patients aged ≥ 18 years old admitted to the FEP Program since 2012 up to April 2024. Only those with a FEP diagnosis were included in this study. Thus, those at clinical high risk (CHR) for psychosis were excluded from this study.

### 2.3 Measures

#### 2.3.1 Sociodemographic and Clinical Variables

Patients' information was collected from the electronic medical records and by consulting patients or the psychiatrist/case manager. At admission to the FEP program, demographic information, including year and place of birth, sex, educational level, residence, housing condition, and marital status was collected. Employment status was defined as being a student (enrolled in any secondary or post‐secondary educational program), employed in a professional occupation, or classified as NEET (Not in Education, Employment, or Training). Clinical information was also collected including the following: Age at psychosis's onset; referrer to the FEP Program; DUP (calculated as the time interval from the onset of at least one positive psychotic symptom to the initiation of antipsychotic medication for treating psychosis, in weeks (Perkins et al. 2005)); diagnosis of psychosis, type of drug formulation; substance use or abuse; previous treatment by the child and adolescent neuropsychiatry service; admission to SerD; number and duration of psychiatric hospitalisation. Information regarding cannabis use was obtained from clinical charts and patient reports. Cannabis use at baseline was defined as any self‐reported or toxicology‐confirmed use during the month preceding admission to the FEP program. Cannabis use at admission was categorised as a dichotomous variable (ever users versus non‐users). Ongoing cannabis use was also recorded at the 1 and 2‐year follow‐up and defined as current use at the time of assessment.

#### 2.3.2 Outcome Measures (HoNOS)

Patients were also assessed with the Italian version of the Health of the Nation Outcome Scale (HoNOS), as recommended by both the local PDTA and the Emilia‐Romagna regional FEP guidelines, at admission and every six months for two years. The HoNOS is a hetero‐administered scale consisting of 12 items, each rated on a Likert scale from 0 (no problem) to 4 (severe or very severe problem) (Preti et al. 2012). As a result, higher scores indicate more severe symptoms and overall clinical presentation. The HoNOS therefore assesses aspects related to both clinical and functional recovery. Patients were considered in recovery if the total HoNOS score was < 8 since this score corresponds to a level of symptomatology that allows the discharge of the patient from the CMHC with a referral to the general practitioner's care (Belvederi Murri et al. 2021; Ferrara, Guloksuz, et al. 2019; Prowse and Coombs 2009).

At 1 and 2 years follow‐up evaluation, outcome indicators were also acquired, including employment status and type of antipsychotic prescription, defined as the use of either oral antipsychotic medication or long‐acting injectable (LAI) formulation.

### 2.4 Statistical Analysis

The total sample was divided into two groups based on cannabis use used in the month preceding the entry into the Program. Participants' characteristics at admission and clinical outcomes were compared between the two groups. Bivariate analyses were conducted to assess any clinical and socio‐demographic differences at baseline between cannabis users and non‐users.

In terms of outcomes, HoNOS scores, employment status and type of antipsychotic prescription were compared between cannabis users and non‐users both at 1‐ and 2‐years follow ups. The same analyses were conducted considering first cannabis status at baseline, then current cannabis use status at each timepoint (1–2 year). The Chi‐square test was used to compare categorical data between cannabis users and non‐users. A student's *t*‐test was used to compare normally distributed numerical data between the two groups. Mann‐Whitney U non‐parametric tests were used for non‐normally distributed variables. Shapiro‐Wilks test was used to detect all departures from normality, rejecting the hypothesis of normality when the *p* value is less than or equal to 0.1.

Finally, Mixed Model for Repeated Measures was used to compare the average HoNOS scores and the recovery rates between the two groups at several endpoints (baseline, 12 months and 24 months). The significance level for all the tests was set at 0.05. Statistical analyses were performed using R v.4.2.1 software.

### 2.5 Ethical Committee and Consent

The local ethical committee (Comitato Etico Area Emilia Centro) with protocol number 761/2021/Oss/AUSLFe approved this study on October 20th, 2021. The study conforms to the principles expressed in the Declaration of Helsinki. Participants, before inclusion in the study, provided informed consent.

## 3 Results

### 3.1 Sample Characteristics at Admission to the FEP Program

A total of 219 patients were admitted to the FEP program between 2012 and April 2024. Of these, 173 met the inclusion criteria and were eligible for the present study. As summarised in Table 1, most participants were male (74.5%), born in Italy (77.4%), living with their parents (83.2%), single (96.5%), without children (97.7%), and had a high school diploma (47.4%). Compared to those without cannabis use (*n* = 90, 52.0%), individuals with cannabis use prior to admission to the program (*n* = 83, 48.0%) were significantly more likely to be male (87.9% vs. 62.2%, *p* < 0.001) and employed (47.0% vs. 25.6%, *p* = 0.003) and less likely to be NEET (not in employment, education or training) (26.5% vs. 41.1%, *p* = 0.04). No significant differences were found between groups regarding nationality, marital or parental status, living arrangements, or educational attainment. Similarly, individuals with cannabis use were slightly younger at both the time of admission to the FEP program (mean age 23.9 vs. 24.7 years, *p* = 0.25) and at psychosis onset (mean age 22.7 vs. 23.1 years, *p* = 0.58), however these differences were not statistically significant.

**TABLE 1:** Demographic and baseline clinical characteristics of the total sample between cannabis users and cannabis non‐users at admission to the program.

	Total *N* = 173	Cannabis users *N* = 83 (48.0)	Cannabis non‐users *N* = 90 (52.0)	*p*‐value
Socio‐demographic characteristics				
Age at program admission, years, mean (SD)	24.3 (4.3)	23.9 (4.0)	24.7 (4.5)	0.25^a^
Age at FEP onset, years, mean (SD)	22.9 (4.4)	22.7 (3.8)	23.1 (4.9)	0.58^a^
5 missing				
Sex, Male, *N* (%)	129 (74.5)	73 (87.9)	56 (62.2)	< 0.001^b^
Born in Italy, yes, *N* (%)	134 (77.4)	67 (80.7)	67 (74.4)	0.32^b^
Marital status				0.50^b^
Single	167 (96.5)	80 (96.4)	87 (96.7)	
Married	3 (1.7)	1 (1.2)	2 (2.2)	
Cohabitant	1 (0.6)	1 (1.2)	0 (0.0)	
Divorced	1 (0.6)	0 (0.0)	1 (1.1)	
Other	1 (0.6)	1 (1.2)	0 (0.0)	
Has children, yes, *N* (%)	4 (2.3)	2 (2.4)	2 (2.2)	0.93^b^
Adopted, yes, *N* (%)	5 (2.9)	3 (3.6)	2 (2.2)	0.58^b^
4 missing				
Community mental health centre, *N* (%)				0.20^b^
Cento	18 (10.4)	9 (10.8)	9 (10.0)	
Codigoro	42 (24.3)	21 (25.3)	21 (23.3)	
Copparo	9 (5.2)	6 (7.2)	3 (3.3)	
Ferrara	81 (46.8)	41 (49.4)	40 (44.4)	
Portomaggiore	23 (13.3)	6 (7.2)	17 (18.9)	
Living situation, *N* (%)				0.59^b^
Temporary accommodation provided by social services	1 (0.6)	1 (1.2)	0 (0.0)	
Other	2 (1.2)	1 (1.2)	1 (1.1)	
Alone or with flatmates	21 (12.1)	9 (10.8)	12 (13.3)	
Homeless	2 (1.2)	2 (2.4)	0 (0.0)	
With parents	144 (83.2)	69 (83.1)	75 (83.3)	
With partner and/or children	3 (1.7)	1 (1.2)	2 (2.2)	
Education level, *N* (%)				0.48^b^
Short‐cycle degree	17 (9.8)	8 (9.6)	9 (10.0)	
Master's degree	6 (3.5)	1 (1.2)	5 (5.5)	
Lower secondary school	64 (37.0)	32 (38.5)	32 (35.5)	
Upper secondary school	82 (47.4)	39 (47.0)	43 (47.8)	
Unknown	4 (2.3)	3 (3.6)	1 (1.1)	
Employment status, *N* (%)				
Student (yes)	55 (31.8)	24 (28.9)	31 (34.4)	0.43^b^
Employed (yes), 2 missing	62 (35.8)	39 (47.0)	23 (25.6)	0.003^b^
NEET (yes)	59 (34.1)	22 (26.5)	37 (41.1)	0.04^b^
Clinical characteristics				
DUP (weeks), mean (SD)	42.6 (90.7)	32.8 (87.6)	52.2 (93.2)	0.05^c^
7 missing				
logDUP (weeks), mean (SD)	2.5 (1.6)	2.2 (1.4)	2.7 (1.7)	0.06^a^
7 missing				
Non‐affective psychosis diagnosis (yes), N (%)	145 (83.8)	65 (78.3)	80 (88.9)	0.09^b^
Referral to FES, *N* (%)				0.45^b^
Relative	4 (2.3)	3 (3.6)	1 (1.1)	
GP	10 (5.8)	3 (3.6)	7 (7.8)	
Psychiatric Ward	86 (49.7)	42 (50.6)	44 (48.9)	
Private specialist	2 (1.2)	2 (2.4)	0 (0.0)	
Community Mental Health Centre	66 (38.2)	31 (37.3)	35 (38.9)	
Other	4 (2.3)	2 (2.4)	2 (2.2)	
Missing	1 (0.6)	0 (0.0)	1 (1.1)	
Antipsychotic, *N* (%)				0.05^b^
No	9 (5.2)	6 (7.2)	3 (3.3)	
Only LAI	20 (11.6)	10 (12.0)	10 (11.1)	
Only Oral	125 (72.2)	53 (63.8)	72 (80.0)	
Both	19 (11.0)	14 (16.9)	5 (5.6)	
				0.04^b^
Only LAI + Both	39 (23.8)	24 (31.2)	15 (17.2)	
Only Oral	125 (76.2)	53 (68.8)	72 (82.8)	
Alcohol abuse, yes, *N* (%)	73 (42.2)	57 (68.7)	16 (17.8)	< 0.001^b^
Tobacco usage, yes, *N* (%)	93 (53.8)	68 (81.9)	25 (27.8)	< 0.001^b^
5 missing				
Received Treatment by Child and Adolescent Neuropsychiatry (yes), *N* (%)	24 (13.9)	8 (9.6)	16 (17.8)	0.12^b^
Admission to SerD (yes), *N* (%)	19 (11.0)	18 (21.7)	1 (1.1)	< 0.001^b^
HoNOS total score (Ahrens et al. 2025; Allegri et al. 2013; Andréasson et al. 1987; Arseneault et al. 2002; Belvederi Murri et al. 2021, 2023, 2025; Berivi et al. 2020; Bloomfield et al. 2014; Carter et al. 2023; Chadwick et al. 2013; D'Souza et al. 2016)	18.3 (6.8)	18.2 (6.8)	18.3 (6.9)	0.91^a^
11 missing				

Abbreviations: DUP, duration of untreated psychosis (calculated as the time, in weeks, from the onset of the first psychotic symptom to the beginning of suitable treatment); FEP, first‐episode psychosis; FES, first‐episode services; GP, General Practitioner; HoNOS, Health of the Nation Outcome Scale; LAI, Long‐acting injectables; NEET, not employed, education and training; SerD, Italian addiction services (Servizi per le Dipendenze Patologiche).

^a^ *t*‐test.

^b^ Chi‐squared test.

^c^ Mann–Whitney test.

In terms of clinical features, the majority of the sample was diagnosed with a non‐affective psychotic disorder (83.8%), and almost half were referred from inpatient psychiatric services (49.7%), with no significant group differences. The median DUP was 42.6 weeks (SD = 90.7), with participants with cannabis use exhibiting a significantly shorter DUP compared to those without cannabis use (32.8 vs. 52.2 weeks, *p* = 0.05).

The mean total HoNOS score at intake was 18.3 (±6.8), indicating moderate severity of illness, no meaningful differences were observed between the two groups.

Compared to those without cannabis use, individuals with cannabis use were significantly more likely to also misuse alcohol (68.7% vs. 17.8%, *p* < 0.001) and to smoke tobacco (81.9% vs. 27.8%, *p* < 0.001), and they had a higher rate of referral to addiction services (SerD) (21.7% vs. 1.1%, *p* < 0.001).

At program entry, the majority of patients (72.2%) were prescribed oral antipsychotic medication. People without cannabis use were more frequently prescribed oral antipsychotics alone (80.0% vs. 63.8%, *p* = 0.05), whereas those with cannabis use more often received a combination of oral and long‐acting injectable (LAIs) antipsychotics (16.9% vs. 5.6%, *p* = 0.05).

### 3.2 Influence of Cannabis Use on Outcomes at 1‐ and 2‐Years Follow‐Up

Tables 2 and 3 summarise the socio‐demographic and clinical characteristics of individuals with cannabis use versus those without cannabis use at the 1‐year and 2‐year follow‐ups, considering cannabis status at baseline and current cannabis status, respectively.

**TABLE 2:** Demographic and clinical characteristics of the total sample, by cannabis status at baseline, at 1‐year follow‐up and 2‐year follow‐up.

	1 year follow‐up				2 year follow‐up			
	Total *N* = 138	baseline Cannabis Users *N* = 68 (49.3%)	baseline Cannabis Non‐Users *N* = 70 (50.7%)	*p*‐value	Total *N* = 104	baseline Cannabis Users *N* = 48 (45.6%)	baseline Cannabis Non‐Users *N* = 56 (54.4%)	*p*‐value
Employment status, N (%)								
Student (yes)	25 (18.1)	8 (11.8)	17 (24.3)	0.05^a^	16 (15.4)	3 (6.3)	13 (23.2)	0.02^a^
Employed (yes)	49 (35.5)	27 (39.7)	22 (31.4)	0.18^a^	48 (46.2)	21 (43.7)	27 (48.2)	0.90^a^
NEET (yes)	35 (25.4)	17 (25.0)	18 (25.7)	0.83^a^	22 (21.4)	12 (25.0)	10 (17.9)	0.22^a^
Antipsychotic therapy, N (%)				0.79^a^				0.66^a^
Only oral	68 (49.3)	30 (44.1)	38 (54.3)		51 (49.0)	22 (45.8)	29 (51.8)	
Oral + LAI	16 (11.6)	9 (13.2)	7 (10.0)		8 (7.7)	5 (10.4)	3 (5.4)	
Only LAI	29 (21.0)	15 (22.1)	14 (20.0)		23 (22.1)	12 (25.0)	11 (19.6)	
None	12 (8.7)	6 (8.8)	6 (8.6)		15 (14.4)	6 (12.5)	9 (16.1)	
Unknown	13 (9.4)	8 (11.8)	5 (7.1)		7 (6.7)	3 (6.3)	4 (7.1)	
				0.44^a^				0.42^a^
Only LAI + Both	45 (39.8)	24 (44.4)	21 (35.6)		31 (37.8)	17 (43.6)	14 (32.6)	
Only Oral	68 (60.2)	30 (55.6)	38 (64.4)		51 (62.2)	22 (56.4)	29 (67.4)	
*None and Unknown excluded								
HoNOS total score, mean (SD)	9.7 (5.8)	9.3 (5.3)	10.1 (6.3)	0.52^b^	8.6 (5.6)	8.5 (5.3)	8.7 (5.8)	0.85^b^

Abbreviations: HoNOS, Health of the Nation Outcome Scale; LAI, Long‐acting injectables; NEET, not employed, education and training.

^a^ Chi‐squared test.

^b^ *t*‐test.

**TABLE 3:** Demographic and clinical characteristics of the total sample, by current cannabis status, at 1‐year follow‐up and 2‐year follow‐up.

	1 year follow‐up 18 NA	2 year follow‐up						
	Total *N* = 120	Currently cannabis users *N* = 38 (31.7%)	Currently cannabis non‐users *N* = 82 (68.3%)	*p*‐value	Total *N* = 89	Currently cannabis users *N* = 18 (20.2%)	Currently cannabis non‐users *N* = 71 (79.8%)	*p*‐value
Employment status, *N* (%)								
Student (yes)	25 (20.8)	6 (15.8)	19 (23.2)	0.36^a^	16 (18.0)	2 (11.1)	14 (19.7)	0.33^a^
Employed (yes)	46 (38.3)	16 (42.1)	30 (36.6)	0.58^a^	46 (51.7)	9 (50.0)	37 (52.1)	0.96^a^
NEET (yes)	32 (26.7)	11 (28.9)	21 (25.6)	0.73^a^	21 (23.6)	5 (27.8)	16 (22.5)	0.61^a^
Antipsychotic therapy, *N* (%)								
Only oral	58 (48.3)	12 (31.6)	46 (56.1)	0.01^a^	47 (52.8)	7 (38.9)	40 (56.3)	0.35^a^
Oral + LAI	14 (11.7)	8 (21.1)	6 (7.3)		7 (7.7)	3 (16.7)	4 (5.6)	
Only LAI	29 (24.2)	8 (21.1)	21 (25.6)		20 (22.5)	5 (27.9)	15 (21.1)	
None	11 (9.2)	6 (15.8)	5 (6.1)		14 (15.7)	3 (16.7)	11 (15.5)	
Unknown	8 (6.7)	4 (10.5)	4 (4.9)		1 (1.1)	0 (0.0)	1 (1.4)	
Only LAI + Both	43 (42.6)	16 (57.1)	27 (37.0)	0.07^a^	27 (36.5)	8 (53.3)	19 (32.2)	0.22^a^
Only Oral	58 (57.4)	12 (42.9)	46 (63.0)		47 (63.5)	7 (46.7)	40 (67.8)	
*None excluded								
HoNOS total score, mean (SD)	9.8 (5.9)	10.3 (5.3)	9.6 (6.1)	0.60^b^	8.6 (5.6)	10.7 (5.8)	8.2 (5.5)	0.19^b^

*Note:* Within the participants reporting ongoing cannabis use, at the 1‐year follow‐up, 36 participants were baseline users and 2 were new users; at the 2‐year follow‐up, all participants reporting ongoing cannabis use were baseline users.

Abbreviations: HoNOS, Health of the Nation Outcome Scale; LAI, Long‐Acting Injectables; NEET = not employed, education and training.

^a^ Chi‐squared test.

^b^ *t*‐test.

At both time points, and for both cannabis status conditions, no significant differences were observed between the groups in terms of overall psychopathology, as assessed by the total HoNOS score.

When cannabis status at baseline was taken into account, participants with cannabis use showed a lower rate of student status at both the 1‐year (11.8% vs. 24.3%, *p* = 0.05) and 2‐year follow‐up (6.3% vs. 23.2%, *p* = 0.02) compared to those without cannabis use. The proportions of employed and NEET individuals were similar between the groups, and there were no significant differences in antipsychotic prescriptions.

Considering current cannabis status at the time of the follow‐up assessments, the only significant difference was shown at the 1‐year follow‐up: Individuals without cannabis use were more frequently prescribed oral antipsychotics (56.1% vs. 31.6%, *p* = 0.05), whereas those with cannabis use more often received a combination of LAI and oral antipsychotics (21.1% vs. 7.3%, *p* = 0.05). Notably, by the 2‐year follow‐up, only 20.2% of the overall sample reported current cannabis use, compared with 48% of users at baseline.

**FIGURE 1:**
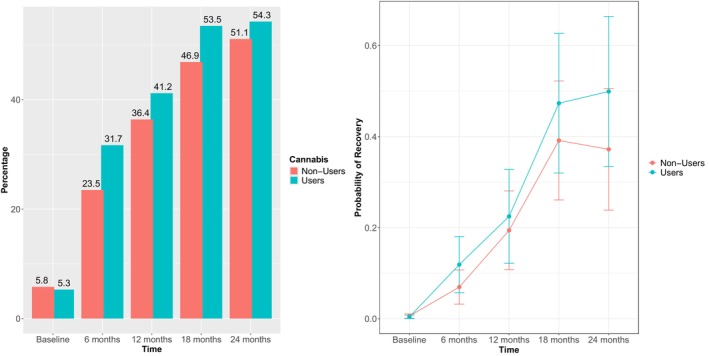
Clinical recovery (HoNOS < 8) in cannabis users compared to non‐users over a 2‐year follow‐up.

Further analyses were conducted to examine how overall symptomatology (HoNOS total score) and recovery status (HoNOS total < 8) evolved overtime in the two groups (Table 4). No significant differences were found between people with cannabis use and those without cannabis use in terms of HoNOS scores or recovery rates at any time point (baseline, 12 months, 24 months) (Supplementary Figure 1). Of note, most patients achieved a recovery status at the 2‐year follow‐up regardless of their cannabis status at entry (54.3% for individuals with cannabis use and 51.1% for those without cannabis use) (Figure 1).

**TABLE 4:** MMRM derived baseline, 6‐month, 12‐month, 18‐month, and 24‐month estimates and 95% confidence intervals and group by time interactions for the outcome measures for the cannabis users versus non‐users.

Outcome	Baseline		*p*‐value^a^	12‐month		*p*‐value^a^	24‐month		*p*‐value^a^	*p*‐value^b^
	Cannabis users	Cannabis Non‐users		Cannabis users	Cannabis Non‐users		Cannabis users	Cannabis Non‐users		
HoNOS tot, 1–12			0.69			0.69			0.45	0.70
mean	18.9	18.5		10.2	10.7		8.8	9.8		
(CI)	(17.3, 20.5)	(17.1, 19.9)		(8.4, 12.0)	(9.1, 12.3)		(6.8, 10.9)	(8.1, 11.5)		
Recovery										0.21
probability (CI)	0.004	0.006	0.72	0.22	0.19	0.82	0.50	0.37	0.54	
	(0.001, 0.02)	(0.001, 0.03)		(0.08, 0.48)	(0.08, 0.41)		(0.21, 0.78)	(0.16, 0.21)		

*Note:* The model is adjusted for sex and age at program admission.

Abbreviation: HoNOS, Health of the Nation Outcome Scale.

^a^ Bonferroni adjustment for multiple comparisons.

^b^ *p* values for the group (cannabis users vs. non‐users) by time interaction effect over the 24 months of treatment.

## 4 Discussion

This longitudinal cohort study investigated the associations between cannabis use prior to admission into a FEP program and clinical outcomes at 1‐ and 2‐year follow‐ups, specifically examining overall symptom severity, functional status, antipsychotic prescription patterns and clinical recovery.

Our findings indicate that at the time of entry into the FEP program, people with cannabis use—compared to those without cannabis use—were more likely to be male, report concurrent use of tobacco and alcohol, and have a prior history of contact with addiction services (SerD). These results are consistent with previous literature, which highlights a higher prevalence of substance use and greater psychiatric comorbidity among males with psychosis (Ochoa et al. 2012; Thorup et al. 2014). This underscores the importance of early, integrated and gender‐sensitive interventions targeting substance use and mental health in youth populations. However, it is worth noting that sex‐related differences in substance use prevalence may be partly influenced by detection biases. Specifically, the underutilisation of routine toxicological screening may lead to an underestimation of substance use among women (Beasley et al. 2014; Kelly et al. 2001). This calls for greater standardisation and equity in assessment practices across sexes in both clinical and research settings (Wierenga et al. 2024).

A second notable finding is that, in contrast with some expectations and previous studies (Quattrone et al. 2021), individuals with cannabis use did not present with an earlier onset of psychosis or more severe psychotic symptoms at baseline. One possible explanation lies in the timing of symptom assessment: In our study, most participants—regardless of cannabis use—were referred to the FEP program following psychiatric hospitalisation, during which they had already received pharmacological treatment. As a result, the clinical presentation at entry to the program may not reflect the severity of the initial untreated psychotic episode, potentially masking baseline differences between people with cannabis use and those without cannabis use. Moreover, the use of the HoNOS as the primary clinical assessment tool may have contributed to an underestimation of symptom severity, as it is not specifically designed to capture the full range or intensity of psychotic symptoms. This limitation could have reduced the sensitivity in detecting baseline differences in psychosis severity between the two groups. Interestingly, individuals with cannabis use did show a significantly shorter DUP at baseline compared to those without cannabis use (32.8 vs. 52.2 weeks). This could be attributed to the predominance of male users with more pronounced behavioural disturbances, such as agitation or disorganisation, which often prompt earlier psychiatric intervention (Ferrara and Srihari 2021). Previous studies have reported similar associations between cannabis use, particularly in young men, and the emergence of disruptive or high‐risk behaviours that lead to quicker clinical detection (Irving et al. 2021; Carter et al. 2023).

Another important main finding is that, contrary to expectations, people with cannabis use did not show a worse prognosis at 1 and 2 years follow‐up in terms of overall clinical psychopathology and recovery rate. Also, Scheffler et al. (2021) led similar research to investigate if cannabis consumption had an impact on outcome in psychoses over 24 months of treatment. They found that the overall severity of psychotic symptoms decreased over the 24 months of treatment, but the initial symptom severity and the symptom reduction were not negatively impacted by cannabis use. This was in contrast with previous studies where they found an association between cannabis use and worse clinical outcome (Foti et al. 2010; Van der Meer and Velthorst 2015). All these studies were conducted using different measures to assess patients' symptoms and clinical outcome: Van der Meer and Velthorst (2015) and Scheffler et al. (2021) used the Positive and Negative Syndrome Scale, while Foti et al. (2010) used the Scale for the Assessment of Positive Symptoms and the Scale for the Assessment of Negative Symptoms. Hence, these discordant results could be due to the lack of a standardised tool to evaluate illness symptoms and illness course in the FEP Program. This limitation could be overcome by adapting a structured and specific illness scale along with the HoNOS evaluation (Ferrara et al. 2024).

Finally, our results showed that participants with cannabis use at baseline and those who were using cannabis at the 1‐year follow‐up—but not at the 2‐year follow‐up—were more likely to receive a combined prescription of depot and oral antipsychotics compared to those without cannabis use. Our findings contribute to a growing body of literature indicating that cannabis use in FEP patients is associated with a possible need to use high dosage or combination of different antipsychotic formulations, which in turn is associated with an increased risk of side effects, such as a higher risk of extrapyramidal symptoms, metabolic consequences, cognitive impairment, and decreased quality of life. Moreover, a landmark study of 2026 patients in South London found that cannabis users at first presentation were prescribed a greater number of unique antipsychotic medications—a proxy for treatment failure—relative to non‐users (Patel et al. 2016). This escalation in antipsychotic changes mediated poorer outcomes, including more frequent and involuntary admissions as well as longer hospital stays. These observational data suggest that cannabis use may diminish treatment responsiveness, necessitating multiple antipsychotic trials. In the south London cohort, the number of unique antipsychotics largely mediated the association between cannabis and higher rates of compulsory admissions and prolonged hospitalisations, highlighting an indirect pathway through antipsychotic inefficacy (Patel et al. 2016). In contrast to these findings, our study did not reveal significant differences in clinical outcomes, as assessed solely through the HoNOS. Notably, our analysis lacked data on rehospitalisations and clinical relapses, which limits the interpretability of our results. It is also worth noting that the difference in antipsychotic prescription patterns between individuals with cannabis use and those without cannabis use observed at baseline was no longer evident at the 2‐year follow‐up. This may be partially explained by the substantial decrease in the proportion of people with cannabis use over time, from 48% at baseline to 20.2% at the 24‐month follow‐up. However, the reduced sample size at follow‐up likely further limited the statistical power of our analyses.

The higher rate of antipsychotic prescriptions observed among individuals with cannabis use, compared to those without cannabis use, may not solely reflect greater clinical severity, but could also be partly explained by lower treatment adherence. Reduced compliance with pharmacological treatment is a well‐documented issue in patients with early psychosis who use cannabis (Foglia et al. 2017; Miller et al. 2009), often leading clinicians to adopt more intensive therapeutic strategies, such as the addition of LAI antipsychotics, in an effort to improve adherence and ensure continuity of care. Lastly, from a mechanistic perspective, these clinical patterns might also be explained by the effects of cannabis on dopaminergic and neuroinflammatory pathways. Cannabis use may, in fact, exacerbate psychotic symptoms by altering dopamine transmission or by contributing to neuroinflammatory processes, which in turn may not only increase the severity of psychosis but also reduce the effectiveness of antipsychotic treatment (Bloomfield et al. 2014; Ahrens et al. 2025).

This study has several notable strengths. First, the sample was carefully characterised using precise and rigorous inclusion criteria. Second, both clinical and socio‐demographic characteristics were examined within a real‐world clinical setting, providing an authentic representation of FEP outcomes. Third, comprehensive clinical assessments were conducted at multiple serial time points using the HoNOS scale, starting from admission. Finally, the study describes outcomes observed within the context of a specialised FEP program, including recovery rates, without implying direct comparison with other care models.

However, some important limitations should be acknowledged. First, the relatively small sample size may have limited the statistical power and affected the significance of some findings. Second, the study's duration contributed to a relatively high attrition rate; notably, patients who were discharged due to full recovery were not assessed at follow‐up, potentially biasing the results. Third, cannabis use was only classified dichotomously using a low exposure threshold (any use in the previous month), resulting in a heterogeneous exposure group likely including occasional users. This may have limited the ability to detect dose–response relationships, particularly given evidence that high‐potency cannabis use is most strongly associated with adverse outcomes. Therefore, the absence of significant differences should not be interpreted as evidence against the clinical relevance of problematic patterns of cannabis use. Fourth, although the study was longitudinal, its observational design precludes causal inferences regarding the associations examined. Fifth, cognitive functioning, including social cognition, was not assessed in the present study. However, emerging evidence, including findings from the EUGEI study, suggests that individuals with psychosis who report cannabis use may exhibit relatively preserved social cognition compared to those without cannabis use (Ferraro et al. 2020), thus possibly influencing both clinical presentation and outcomes. This aspect may partly contribute to the absence of observed differences in functional outcomes and warrants further investigation. Lastly, several clinically relevant factors that could influence outcomes, such as treatment adherence (both pharmacological and psychosocial), data on rehospitalisations, family support, and comorbid psychiatric conditions (e.g., personality disorders), were not assessed and warrant further investigation (Berivi et al. 2020).

## 5 Conclusion

These findings highlight the need for early detection of cannabis use, motivation‐enhancing interventions, and integrated care models combining psychiatric treatment with substance use counselling. While cannabis use prior to program entry was not associated with significant differences in overall clinical outcomes in our sample, addressing cannabis use remains clinically relevant to reduce treatment burden, support treatment adherence, and account for the distinct sociodemographic and substance‐use profiles of cannabis users. Future randomised trials should evaluate whether reducing cannabis use can decrease antipsychotic load and improve long‐term outcomes. Neurobiological studies are warranted to elucidate biological mechanisms—particularly the interactions between cannabinoids, dopamine receptor sensitivity, and neuroinflammation—that might influence FEP outcomes.

## Funding

This work was supported by NEXTGENERATIONEU (NGEU) and funded by the Ministry of University and Research (MUR), National Recovery and Resilience Plan (NRRP), project MNESYS (PE0000006) – A Multiscale integrated approach to the study of the nervous system in health and disease (DN. 1553 11.10.2022) (D.D.D., M.B.V., L.G., and M.F.).

## Conflicts of Interest

The authors declare no conflicts of interest.

## Supporting information


**Figure S1:** Least Square Means of HoNOS total score over 24 months of follow‐up in cannabis users and cannabis non‐users.

## Data Availability

The data that support the findings of this study are available on request from the corresponding author. The data are not publicly available due to privacy and ethical restrictions.
