# Supplementary material for: Cannabis Use and Clinical Outcomes in First Episode Psychosis: Results From a 2‐Year Follow‐Up Study
Source: Early Interv Psychiatry. 2026 May 16;20:e70179. doi: 10.1111/eip.70179 (PMC13179528; doi:10.1111/eip.70179)
Supplement: Supplementary file 1 — Figure S1: Least Square Means of HoNOS total score over 24 months of follow‐up in cannabis users and cannabis non‐users. [file EIP-20-0-s001.docx]

Supplementary Material

**Fig. S1** Least Square Means of HoNOS total score over 24 months of follow up in cannabis users and cannabis non-users.
